# Guidelines for benchmarking of optimization-based approaches for fitting mathematical models

**DOI:** 10.1186/s13059-019-1887-9

**Published:** 2019-12-16

**Authors:** Clemens Kreutz

**Affiliations:** 1 Faculty of Medicine and Medical Center, Institute of Medical Biometry and Statistics, University of Freiburg, Stefan-Meier-Str. 26, Freiburg, 79104 Germany; 2 CIBSS–Centre for Integrative Biological Signalling Studies, University of Freiburg, Freiburg, Germany

**Keywords:** Benchmarking, Differential equation models, Optimization, Parameter estimation, Systems biology

## Abstract

Insufficient performance of optimization-based approaches for the fitting of mathematical models is still a major bottleneck in systems biology. In this article, the reasons and methodological challenges are summarized as well as their impact in benchmark studies. Important aspects for achieving an increased level of evidence for benchmark results are discussed. Based on general guidelines for benchmarking in computational biology, a collection of tailored guidelines is presented for performing informative and unbiased benchmarking of optimization-based fitting approaches. Comprehensive benchmark studies based on these recommendations are urgently required for the establishment of a robust and reliable methodology for the systems biology community.

## Introduction

A broad range of mathematical models is applied in systems biology. Depending on the questions of interest and on the amount of available data, the type of models and the level of detail vary. Most frequently, *ordinary differential equation models (ODEs)* are applied because they enable a non-discretized description of the dynamics of a system and allow for quantitative evaluation of experimental data including statistical interpretations in terms of confidence and significance. In the *BioModels Database* [1], currently, 83% of all models which are uniquely assigned to a modeling approach are ODE models. In this article, I focus on the optimization-based fitting of these models although many aspects are general and also apply to other modeling types and approaches.

Typical parameters in systems biology such as the abundances of compounds or the strengths and velocities of biochemical interactions are typically context-dependent, i.e., they vary between species, tissues, and cell types. Hence, they are represented as unknown parameters in mathematical models. Application-specific calibration of the models is therefore required which corresponds to the estimation of these unknown parameters based on experimental data.

In most cases, parameter estimation is performed by the optimization of a suitable *objective function* such as minimization of the sum of squared residuals for *least squares* estimation or maximization of the likelihood for *maximum likelihood estimation* [2]. Both approaches coincide with normally distributed measurement errors. Such optimization-based fitting of a model requires the selection of a generic numerical optimization approach as a core algorithm. In addition, the optimization problem needs to be defined in terms of initialization, search space, termination criteria, and *hyperparameters* that set up and configure the numerical algorithms. Although parameter estimation is a central task of modeling, the lack of reliable computational approaches for fitting is still a bottleneck in systems biology. The absence of high-performing software implementations seems to be a major reason why ODE-based modeling is not yet a routinely applied computational approach for analyzing experimental data.

The importance of proper designs for benchmark studies in computational biology has been discussed in several publications [3–5]. General guidelines have been provided recently for computational analysis of omics data [6], multiple alignment of protein sequences [7], and supervised classification methods [8] as well as for periodic scientific benchmarking [9] and general studies in computational biology [10–12]. In this article, these aspects are discussed in the context of benchmarking of approaches for optimization-based fitting of mathematical models in systems biology. For simplicity, in the following, this task is sometimes briefly denoted as benchmarking of optimization approaches, although it always refers to the optimization for the fitting of parameters of ODE models which is also termed calibration or model calibration in the literature.

## Why is reliable fitting challenging?

A major characteristic of the mathematical models applied in systems biology is the intention to mirror the biological process of interest because this facilitates enhanced possibilities of interpretations and understanding. To this end, molecular compounds such as proteins and spatial compartments such as cells are defined as model components. Moreover, biochemical interactions between the considered compounds are translated as rate equations into the model. In contrast to *phenomenological* models which describe the empirical relationships in an abstract and simplified manner, the complexity of these so-called *mechanistic* models is dictated by the complexity of the investigated biological process.

Estimating the parameters of typical models in systems biology requires data that covers a broad set of experimental conditions such as multiple time points, genetic perturbations, and/or treatments. Since the evaluation of distinct experimental conditions is elaborate, the amount of available experimental data for parameter estimation is always limited. In such kind of settings, multiple parameter combinations can give rise to the same model response for experimentally investigated conditions. A measured steady-state concentration, for instance, might merely provide information about the ratio *k*_prod_/*k*_deg_ of production and degradation rates. Because different combinations of the individual parameters *k*_prod_ and *k*_deg_ result in the same steady state, all combinations with the same ratio fit the data equally well. Such sub-spaces where the objective function is entirely flat has been termed *non-identifiability*. Because non-identifiability is common features of models in systems biology, the equations which have to be solved during optimization are ill-conditioned or even have non-unique solutions which decreases the performance of numerical algorithms.

The following typical attributes of mechanistic models in systems biology raise methodological challenges for the model fitting:

- The models are large in terms of the number of parameters and dynamic variables. Thus, optimization has to be performed in high-dimensional spaces.
- Fast and robust numerical optimization algorithms do not exist for general non-linear problems. Since the objective functions used for the fitting depend on the model parameters (strongly) non-linearly, numerical optimization is intricate and local optima may exist.
- The evaluation of the objective function comprises the numerical integration of the ODEs which is computationally demanding and only feasible with limited numerical accuracy.
- Because explicit solutions *x*(*t*) for the ODEs $\dot x = f(x)$ cannot be derived by analytical calculations, all mathematical calculations requiring *x*(*t*) in an explicit form are infeasible.
- Derivatives of the objective function have limited numerical accuracy and cannot be calculated naively.
- Parameter values vary over several orders of magnitudes, and usually, only a limited amount of prior knowledge is available. Therefore, it is difficult to specify priors, initial guesses, and/or bounds.
- Optimization has to cope with constraints like upper and lower bounds and with non-identifiability, i.e., with ill-conditioning and entirely flat sub-spaces.

In addition, discontinuities of external inputs (so-called *events*) [13] might occur which has to be handled properly. Sometimes, steady-state constraints for the initial values have to be implemented numerically which is another source of performance loss [14].

## Existing approaches and benchmark studies

**Fig. 1:**
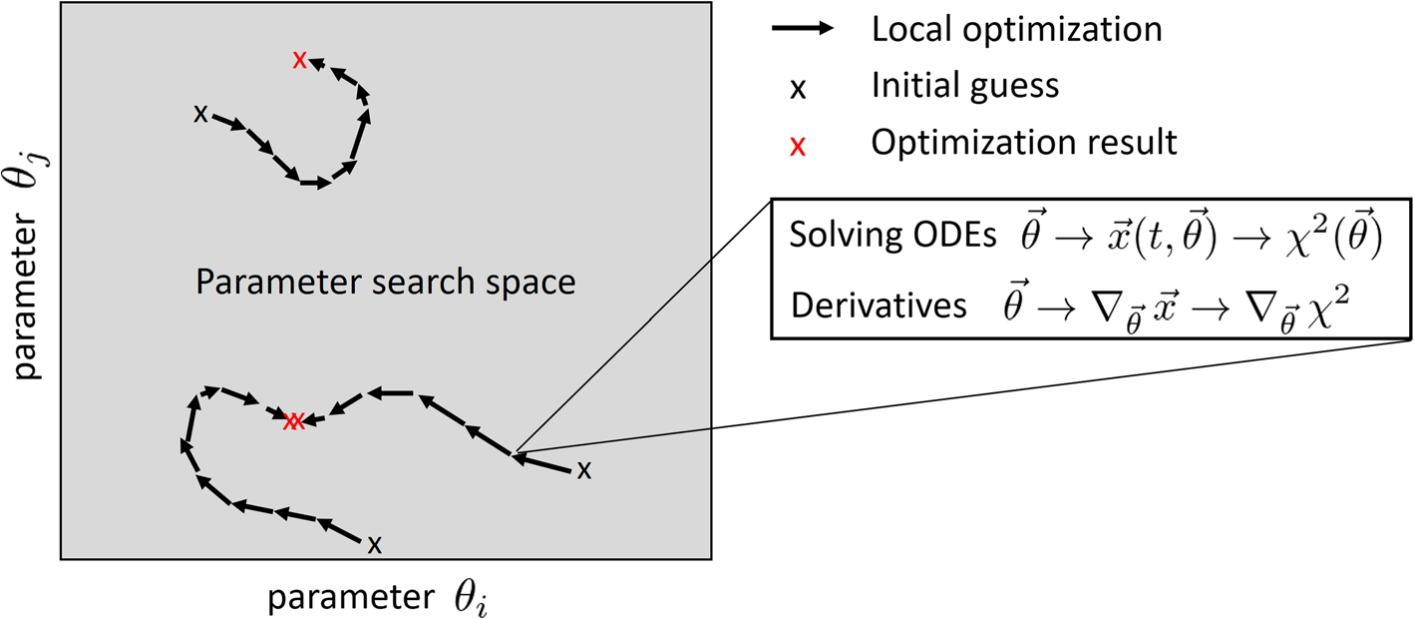
Tasks to be accomplished for fitting ODE models. The fitting of ODE models requires several generic tasks. The optimization problem has to be defined in terms of bounds of the search space and geometry (e.g., linear vs. log scale). Moreover, the selected generic optimization algorithm applied as the core of optimization-based fitting has to be initialized. There are many ways of combining global and local search strategies. A prominent global search strategy is random drawing of multiple initial guesses and performing local optimization for each starting point. In each optimization step of a local optimization run, the ODEs have to be solved for the evaluation of the objective function *χ*^2^(*θ*). Incremental improvement strategies are applied for suggesting a new parameter vector for the next iteration step in the core optimization routine which is usually performed based on derivatives or approximations thereof

Fitting ODE models requires the combination of several generic tasks as indicated in Fig. 1. For these individual tasks, a multitude of methods have been published (e.g., as summarized in [2, 15, 16]). For a given parameter vector, the ODEs have to be solved by numerical ODE integration methods in order to evaluate the objective function. Moreover, iterative optimization requires a strategy for suggesting the next trial parameters in each optimization step. For this, derivatives have to be calculated or approximated, or incremental improvement has to be obtained by an alternative approach. Such iterative local optimization approaches are typically deterministic and are usually combined with a stochastic global search strategy in order to enable convergence to the global optimum. For optimization-based fitting, these generic tasks have to be combined reasonably in order to obtain a robust and reliable approach. However, existing benchmark studies provide only a heterogeneous, fragmentary, and partly inconsistent picture about the performance and applicability of such model calibration approaches and how the different numerical tasks are combined most efficiently.

An early publication in systems biology found superior performance of the so-called *multiple shooting* [17] for local optimization. The idea behind multiple shooting is to reduce the non-linear dependency on the parameters by partitioning time courses into multiple short intervals. Yet, this method is difficult or even impossible to apply for partly observed systems with complex observation functions. It requires a custom implementation of an ODE integration method, and its combination with global search strategies has not been considered. Moreover, there are no implementations publicly available. Consequently, this methodology disappeared from the systems biology field during recent years.

Repeating deterministic optimization runs for multiple initial guesses, i.e., so-called multi-start optimization, showed superior performance in the *Dialogue for Reverse Engineering Assessments and Methods (DREAM)* benchmark challenges about parameter estimation [18] and network reconstruction [19]. Here, a *trust region* and gradient-based deterministic non-linear least squares optimization approach [20] has been utilized as a local optimization strategy, and a global search was performed by utilizing multiple runs with random initial guesses. Trust regions are iteratively updated confidence areas indicating sufficient quality of the local approximation of the objective function which yields successful optimization steps. This approach is implemented in the *Data2Dynamics* modeling framework [21] and proved to be superior to other approaches in several studies [22–24].

It has been shown that deterministic gradient-based optimization is superior to a batch of stochastic algorithms and hybrid approaches which combine deterministic and stochastic search strategies [22]. In contrast, other studies found superior performance of stochastic optimization methods [25–28]. It was confirmed that multi-start gradient-based local optimization is often a successful strategy [29], but on average, a better performance could be achieved with a hybrid metaheuristic [25] combining deterministic gradient-based optimization with a global scatter search metaheuristic.

Finite differences (*F*(*θ*+*h*)−*F*(*θ*))/*h* with step size *h* are the most naive way of calculating a derivative of an objective function *F*(*θ*) with respect to parameter *θ*. While it has been shown that derivative calculation based on finite differences is inappropriate for ODE models [22, 30], this outcome has been partly questioned [31], at least if optimization is performed on a normalized data scale. In addition, for large models, the so-called *adjoint sensitivities* were reported to be computationally most efficient for derivative calculations [30].

It has been repeatedly stated that parameters are preferably optimized on the log scale [5, 29, 32]. Nevertheless, optimization of model parameters is still frequently performed at the linear scale in applications and even in benchmark studies [31].

In addition to neutral publications that focus primarily on benchmarking, there are others that are geared towards introducing a new approach while comparing the performance of alternative approaches less comprehensively. Despite this rather large amount of studies, there is currently no consensus and there are no clear rules in the systems biology community regarding the proper selection of approaches for the parameter fitting. This demonstrates that to date, benchmark studies lack convincing evidence and reveals the need for improved benchmark analyses.

## Pitfalls of benchmark studies

### P1: Unrealistic setup

In order to be able to draw valid conclusions for real application problems, benchmark studies have to restrict to identical settings and the same amount of information as available in real application settings. In our context, simulated data deviate from this requirement because real experimental data in molecular biology typically contain non-trivial correlations, artifacts, or systematic errors which is usually not considered for simulated data sets. Simulating data for assessing model calibration approaches requires much more specifications than in most other fields of computational biology. The reason for this is that realistic combinations of sampling times, observables, observation functions, error models, and experimental conditions have to be defined because this has a great impact on the amount of information provided by the data. Moreover, in order to successfully reject incomplete model structures, it is necessary that the fitting of experimental data works for such wrong models as well. Since simulated data is typically generated with the same model structure used for fitting, there is usually no mismatch between the model and the data. Thus, optimization is only evaluated for settings where a correct model structure is available. In order to not rely on such critical assumptions, it is highly preferable to assess fitting approaches based on real experimental data.

The common advantage of simulated data is the inherent knowledge about the underlying truth because this usually allows for appraisal in terms of true/false positives or in terms of bias and variance. In contrast to most other benchmarking fields, this aspect is hardly relevant for benchmarking of optimization approaches because, on the one hand, each simulated data realization has a different, unknown optimum. Thus, the global solution is unknown even for simulated data. On the other hand, assessment by distance of estimated and true parameter values is not meaningful because of non-identifiability. Moreover, the outcomes of several optimization approaches can be assessed by evaluating the objective function which is feasible without any restriction for experimental data.

In real applications, there is only limited information available for initialization and for tuning of hyperparameters, for instance, integration tolerances or thresholds defining termination criteria of iterative optimization. Therefore, default configurations have to be utilized or hyperparameters have to be defined by consistency checks. In benchmark studies, one has to strictly avoid tuning of such configurations based on the performance criteria in order to prevent unrealistic performance assessment. Instead, it should be prespecified before the evaluation of the methods of how hyperparameters will be chosen based on the information that is also available in practice. Moreover, tuning of hyperparameters has to be counted as an additional runtime.

### P2: Ignoring covariates

In statistics, *covariates* denote all attributes which can have an impact on the results but are not of primary interest of a study. Improper consideration of relevant covariates is a common and well-known general problem in statistics.

In our setting, the performance comparison of generic optimization approaches as the core of the fitting process is usually of primary interest. These approaches are typically available as different generic algorithms. However, there are a lot of decisions which have to be made for applying those generic algorithms in the context of model calibration and thus appear as covariates: First, a set of benchmark problems has to be selected. Moreover, a strategy for combining global and local search has to be specified, and an ODE integration method has to be selected. In addition, parameter bounds and parameter scales (linear vs. logarithmic) have to be defined and ODE integration algorithms and tolerances have to be specified as well as stopping the criteria for iterative optimization. Each of these decisions should be considered as a covariate.

**Fig. 2:**
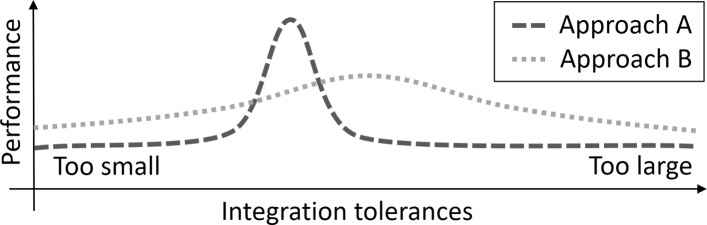
Impact of hyperparameters. Multiple configurations can have an impact on performance. In this illustration example, two optimization approaches have different sensitivities with respect to the choice of tolerances controlling the numerical error of ODE integration. Moreover, both approaches have different optimal choices for this hyperparameter. For most integration tolerances, approach B is superior. However, approach A displays the overall best performance for optimally chosen tolerances. This illustration example highlights the importance of the evaluation of hyperparameters for drawing valid conclusions

The fact that these covariates affect the performance of individual core routines for optimization poses a severe problem. Wrong conclusions can be drawn since it is difficult to entirely uncover the origin of observed performance differences. In the example depicted in Fig. 2, approach A requires properly tuned tolerances controlling the accuracy of ODE integration. If properly chosen, this approach is superior. In contrast, approach B is less sensitive to these tolerances and outperforms A for most choices although the approach can never reach the maximal performance possible for approach A. This example illustrates that observing a performance benefit of one approach for a specific tolerance merely provides a fragmentary and possibly misleading picture.

Analyzing multiple effects by a multi-variate statistical terminology is not yet common for benchmark studies, but well-established, e.g., for clinical studies where patient-specific covariables such as gender, age, and smoking occur as covariates. Table 1 summarizes 15 typical covariates for optimization benchmark studies and illustrates that many decisions have to be made for the implementation of an optimization-based parameter estimation approach.

**Table 1:** Covariates

Abbreviation	Covariate	Typical possible choices
C1	Application problem	Model equations and the data set(s)
C2	Primary performance criteria	Convergence per computation time, iteration steps
C3	Secondary performance criteria	Documentation, user-friendliness, code quality
C4	Parameter scale	Linear vs. log scale
C5	Global search strategy	Multiple initial guesses, scatter search algorithms, stochastic search
C6	Initial guess strategy	Fixed vs. random, normally distributed vs. uniform vs. latin-hypercube
C7	Parameter constraints	Upper and lower bounds
C8	Prior knowledge	None vs. (weakly) informative priors
C9	ODE integration implementation	SUNDIALS, Matlab, R
C10	ODE integration algorithm	Stiff vs. non-stiff approaches, Adams-Moulton vs. BDF
C11	Integration accuracy	ODE integrator tolerances
C12	Derivative calculation	Finite differences, sensitivity equations, adjoint sensitivities
C13	Stopping rule	Optimization termination criteria
C14	Handling of non-converging ODE integration	Termination of optimization vs. infinite loss
C15	Algorithm-specific configurations	Cross-over rate, annealing temperature, number of particles

The performance of an optimization approaches depend on many decisions and configurations C1–C15. For the comparison of several approaches, these attributes appear as covariates. Performance benefits for individual choices do not necessarily indicate a general advantage because benefits might merely originate from the chosen configurations

For the illustration example in Fig. 2, one could determine the impact of the integration tolerance as a covariate by the evaluation of the whole range in a benchmark study. This strategy is feasible in real application settings for a single or very few covariates. However, since the number of combinations increases exponentially, the evaluation of the full configuration space is not feasible for all relevant covariates. Nevertheless, the robustness of the conclusions with respect to the covariates has to be addressed, and limitations of the scope of the conducted studies should be kept in mind. A pragmatic way to achieve this is by verifying that changes of the individual covariates do not alter the outcome dramatically. Moreover, classical study design principles such as balancing and/or randomization can be applied in order to minimize the impact of covariates [5]. As an example, one could randomly select many reasonable choices of all the covariates summarized in Table 1. To prevent bias due to imbalanced random drawings, one could draw the covariates jointly for all studied optimization algorithms to ensure that all optimization algorithms are evaluated for the same set of covariates.

### P3: Performing only case studies

The performance of optimization approaches depends on the application problem, i.e., on the models and the data sets which are used for benchmarking. A chosen benchmark model determines the intricacy of the optimization task in terms of dimension (number of parameters), non-linearity, ill-conditioning, local optima, amount of information provided by the data, etc. and thereby affect the performance. Thus, the choice of application problems can be interpreted as an additional covariate that exhibits a strong effect on optimization performance.

A study where only a single model is evaluated for benchmarking of multiple approaches provides only a minor evidence since the behavior of the applied approaches might be completely different for another application problem. In terms of evidence, such a benchmark study constitutes merely a *case report*. Therefore, benchmark studies have to be performed based on a comprehensive and representative set of test problems in order to draw generally valid conclusions.

### P4: Non-convergence and local optima are hardly distinguishable

Sub-optimal convergence behavior is difficult to be discriminated from local optima since in both cases, the optimization terminates at distinct points in the parameter space, usually with different objective function values. In high-dimensional spaces, it is difficult to evaluate whether a point in the parameter space is a local optimum, especially if the objective function and its derivatives can only be evaluated with limited numerical accuracy. Existing approaches which could be applied like the profile likelihood [33], reconstruction of flat manifolds [34], identifiability analysis [35], or methods based on a local approximation of the curvatures do not enable reliable classification in case of convergence issues and/or numerical inaccuracies. Thus, convergence issues can easily be confused with local optima. New methods for proving local optimality and for detecting convergence issues would be very valuable for the assessment and for future improvements of optimization approaches.

**Fig. 3:**
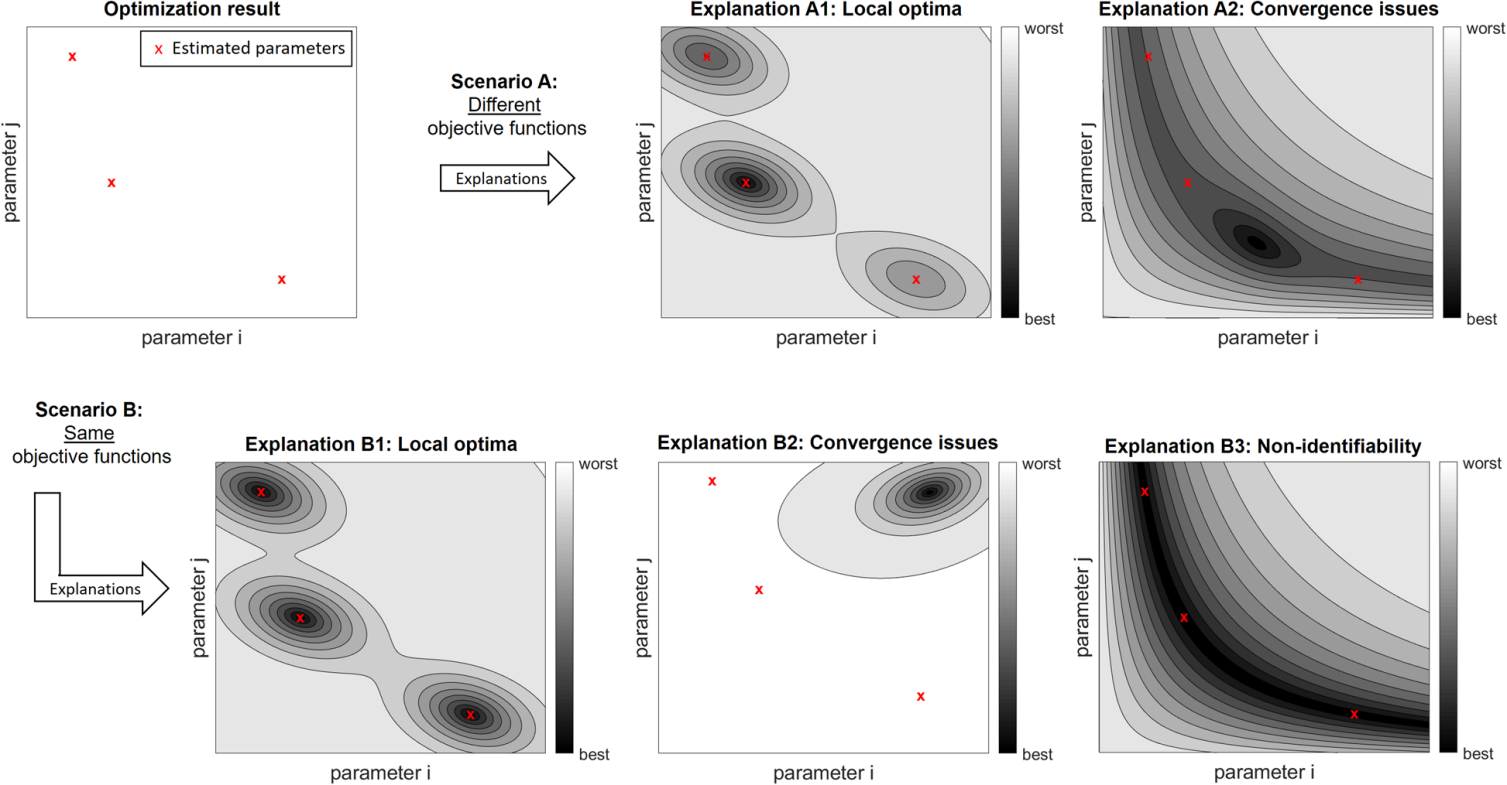
Ambiguous interpretation of optimization outcomes. For non-trivial optimization problems, the results of independent optimization runs are typically not the same. The upper left panel indicates an outcome for three optimization runs, e.g., generated with different starting points. If the objective function values after optimization are different (scenario A), such an outcome could be explained by local optima (explanation A1) or by convergence problems of the optimization algorithm (explanation A2). If the same values are obtained for objective function, there might be several local optima with the same value of the objective function (explanation B1), there might be a convergence problem (B2), or non-identifiabilities might exist (B3), i.e., the estimated parameters are not uniquely specified by the data and then the same value of the objective function is achieved in multi-dimensional sub-spaces

The upper left panel in Fig. 3 shows a hypothetical outcome of three optimization runs. “Scenario A” indicates that both convergence problems and local optima might generate the same observations. Even if identical values for the objective function are obtained (scenario B), the interpretation is ambiguous because there are no reliable approaches to distinguish convergence issues from local optima. Moreover, the third possible explanation in this scenario is associated with non-identifiabilities, i.e., optimal sub-spaces. If non-identifiabilities exist, even high-performing optimization approaches converge to distinct points in the parameter space. For benchmark analyses, this means that it is not reasonable to measure the similarity of the outcomes of multiple optimization runs by distances in the parameter space.

## Guidelines for benchmark studies

I recommend that the guidelines presented below are discussed in future publications in a point-by-point manner in order to provide a summary for the readers about the design of a benchmark study and the resulting evidence in an easily accessible and clearly structured manner. Multiple guidelines might be required to prevent a pitfall. Conversely, an individual guideline might help to prevent several pitfalls.

General guidelines for benchmark studies in the whole computational biology field have been recently presented [12]. Here, I concretize what those general suggestions mean for benchmarking of parameter optimization approaches and discuss their relevance, feasibility, and differences in our context. The terminology and order are kept as similar as possible to [12].

### G1: Clear definition of aim and scope

Benchmark studies which are performed to illustrate the benefits of a newly presented approach are easily biased because they are performed to confirm merits. Often, application problems are utilized where existing methods have limited performance. Consequently, it should be precisely stated whether benchmark analyses are performed for introducing a new approach, or whether the goal is performing a neutral and comprehensive study based on previously published computational approaches and test cases [5, 12].

Since the performance of optimization routines is context-specific, i.e., depends on the chosen benchmark problem, it is essential to define the scope of a study and select representative test cases according to this definition [5]. For mathematical modeling in the systems biology field, one could define the scope by the biological background. Models of signaling pathways, for instance, usually describe the dynamics of activation after stimulation and show transient dynamic responses. In contrast, metabolic models usually constitute steady-state descriptions. Gene regulatory networks, on the other hand, typically have distinct rate laws because activatory and inhibitory effects are described by products of *Hill equations*, instead of *mass action kinetics*. Other options for defining scopes could be based on model attributes like the amount of data, number of parameters, existence of events, or steady-state constraints, or based on dynamic characteristics such as the occurrence of oscillations.

### G2: Inclusion of (all) relevant methods

In contrast to other fields of computational biology, it is currently infeasible to include all relevant optimization methods in a benchmark study because published optimization methods are only available in distinct software or programming environments. In addition, optimization-based fitting of ODE models requires the combination of several numerical tasks which do not perfectly coincide in different programming languages or software package. As an example, the trust region-based non-linear least square optimization approaches implemented in Matlab and R are not identically programmed and produce different outcomes with distinct performances [36].

Moreover, there is not yet an established standard for defining an optimization problem comprehensively including all model equations, data, measurement errors, priors, constraints, algorithms, and hyperparameters. Thus, it is very elaborate and partly infeasible to include approaches which are previously applied in other benchmark studies. This limitation demands for standardized data and documentation formats like *COMBINE* archives [37]. Nevertheless, including as many approaches as possible is an important and indispensible aim. For reasonable interpretation of observed performances, it is absolutely essential to implement at least one state-of-the-art approach and to verify that the implementation or the observed performance is coinciding with previous benchmark studies.

### G3: Selection of realistic and representative test cases

Because the performance of optimization approaches strongly varies between the application models, a rather large number of models is required to obtain a representative and comprehensive set of test cases. However, the number of available benchmark problems is very limited so far. Six benchmark models have been published in [38]; however, four of them only contain simulated data. Recently, a set of 20 benchmark problems with experimental data sets has been published [32], but it still remains difficult to perform larger benchmark studies. Because of the small number of available test cases, it is currently only hardly feasible to define a narrow scope of such a study while still including enough test problems.

Simulation of data is valuable for understanding and validation of an observed performance loss in benchmark studies. However, as argued above as pitfall P1, simulated data has only limited value for benchmarking of optimization-based fitting approaches. Hence, benchmark studies based on experimental data are more representative and thus are strongly recommended.

### G4: Appropriate hyperparameters and software versions

Assessment of fitting approaches requires many decisions at the level of the selection of approaches for the various numerical tasks (e.g., for ODE integration, derivative calculations, global and local search heuristics) as well as on the level of numerical hyperparameters (see Table 1). As discussed above as pitfall P2, these configurations of the optimization approaches commonly strongly impact the performance. Some optimization approaches, for instance, have weak performance if the bounds are reached during optimization and the performance therefore critically depends on the initialization. Therefore, the strategy for the choice of all configurations (including hyperparameters and software versions) has to be pre-specified, and it has to be checked carefully that individual optimization approaches are not privileged in order to guarantee fair comparisons.

To the best of my knowledge, existing differences between subsequent versions of the same software package as well as their impact have not been investigated yet. Nevertheless, in order to guarantee full reproducibility, it is essential to comprehensively describe the applied approach including software versions. Software tools for enhancing the reproducibility of computational analyses have been summarized previously [12].

### G5: Evaluation in terms of key quantitative metrics

Optimization is in almost all circumstances assessed by means of convergence, i.e., in terms of probabilities or frequencies of finding local or global optima. Although all local minima with statistically valid objective function are of interest, commonly, the primary target is revealing the global optimum. Nevertheless, for proving the applicability and testing performance, it is usually also valid to consider convergence to any kind of optimum because whether the global or a local optimum is identified is often only a question of the chosen initial guess and strongly depends on the size of the search space. For deterministic optimization, for instance, each optimum has a region of attraction. Hence, the frequency of finding the global optimum relative to the frequency of converging to a local optimum is mainly a question of the size of the search space and the location of the optimization starting points.

Computational efforts have to be distributed among global and local search strategies, e.g., computational efforts can be spent either for an increasing number or for increasing lengths of the individual optimization runs. In order to balance this trade-off, it is a very reasonable strategy to assess the convergence to local/global optima by calculating the expected runtime for a single converged run as was recently suggested [29]. It should be kept in mind, however, that runtimes are also dictated by the computer system and especially by the degree of parallelization. Thus, one has to ensure that the rating of runtimes is fair. Moreover, it should be investigated whether outcomes depend on the way of parallelization, i.e., on the number of processors.

An additional issue is the definition of convergence which is typically done based on thresholds for the objective function relative to the overall best known solution. The choice of the threshold is a covariate and it is important to investigate its impact. Furthermore, the order of magnitude has to be chosen properly, i.e., to guarantee that only the fits which are in statistical agreement with data and measurement errors are counted as successfully converged.

### G6: Evaluation of secondary measures

The quality of documentation, user-friendliness, and code quality are important for diminishing the risk of incorrect application of optimization approaches and thus are valuable as secondary measures.

Other secondary measures mentioned in the literature are only subordinately relevant since bad convergence behavior cannot be compensated by other aspects. Moreover, traditional trade-offs, e.g., between precision and recall or between bias and variance, do not apply when assessing convergence of optimization algorithms.

Within a model calibration approach, there are many aspects and details which have to match with each other comprehensively. Therefore, algorithm development requires expert knowledge, and traditional strategies for tuning optimization approaches should be exploited until a sufficient convergence behavior is obtained instead of implementing a weakly tested custom solution. In contrast to other fields, the feasibility of user adaptation is therefore not a secondary aim. From my perspective, custom heuristics are not recommended and should not be included in benchmark studies.

### G7: Interpretation and recommendation

The fitting of ODE models requires the combination of several numerical tasks. Optimization only works reliably, if all these components match together and perform sufficiently well. Proper interpretation thus requires the evaluation of the impact of all configuration options (see the “P2: Ignoring covariates” section), and the effect of these covariates has to be considered, for instance, by multi-variate analysis of the performances [5].

Benchmark studies should intend to provide recommendation rules about the selection of optimization approaches for specific scopes of application as explicit as possible, e.g., by deriving decision trees such as “use approach A in case X, use B otherwise”.

An advantage of benchmark studies in this field is that multiple optimization approaches can be applied subsequently in order to optimize the objective function. One could therefore apply multiple well-performing approaches consecutively if explicit rules for selecting single approaches cannot be derived.

### G8: Publication and reproducible reporting of results

For optimization, small technical details might deteriorate performances and thereby strongly impact the outcomes of benchmark studies. Thus, it is critically essential to publish the source code of the performed analyses as well as details and versions of programming environments, operating systems, and software packages. This also permits to subsequently extend the benchmark study by additional optimization approaches as well as new test problems. Thereby, continuous updates and refinements are enabled that prevents studies from getting outdated.

## Conclusions

Several methodological challenges appear for optimization-based parameter fitting of ODE models. The available benchmark studies indicate that ODE models from the systems biology field demand such a variety of methodological requirements that each optimization approach is prone to failing. Unfortunately, existing studies provide only a fragmentary and inconsistent picture about the applicability of existing approaches, and there is no consensus about the proper selection of optimization approaches in the systems biology field. Thus, reliable fitting of mathematical models remains a limiting bottleneck in systems biology.

In this article, four major pitfalls for the design, analysis, and interpretation of benchmark studies have been discussed. Moreover, general guidelines from the literature were tailored to the optimization-based parameter estimation setting. The presented pitfalls and guidelines indicate conceptional needs: Standards for exchanging results of model analyses have to be improved in order to permit comparisons over multiple software environments. In addition, approaches for discriminating between numerical convergence issues and local optima have to be established. Moreover, there is an urgent need for further realistic benchmark questions and challenges.

In order to obtain consensus within the community, it seems a very promising strategy to make the entire fitting environments available for the community, e.g., as online tools where users can upload their models and data. This would also guarantee the reproducibility of performance assessment studies and enable future extensions by new optimization methods or additional benchmark problems.

## Supplementary information

**Additional file 1** Review history.
