# Supplementary material for: Guidelines for benchmarking of optimization-based approaches for fitting mathematical models
Source: Genome Biol. 2019 Dec 16;20:281. doi: 10.1186/s13059-019-1887-9 (PMC6915982; doi:10.1186/s13059-019-1887-9)
Supplement: Supplementary file 1 — Additional file 1 Review history. [file 13059_2019_1887_MOESM1_ESM.docx]

Review History

**First round of review**

**Reviewer 1**

**Is the topic of the article timely and of interest to a wide range of readers?**

Yes

**Is the article well written and presented in a logical way?**

No: The paper is not very clean.

**Do the authors cover the relevant literature in an accurate and balanced way?**

Yes

**Do the authors provide a useful synthesis of the topic?**

Yes

**Do the authors provide insightful discussion of the future directions for the field?**

Yes: I hesitated with "no". The discussion is ok, but not very good.

**Do any tables, figures or boxes that are present enhance the manuscript?**

Yes

**Would the manuscript benefit from any additional tables, figures or boxes?**

Yes

**Comments to author:**

This paper handles an important topic and fits well into the current "benchmarking wave" in Genome Biology. I however have two major concerns:

- The proposed rules are, as admitted by the author himself, strongly related with the rules presented in the recent paper by Weber et al. Some repetition may be ok, but here I feel that this is too much. A possibility to avoid repetitions would be to generally formulate the current paper in the vein of "what do the Weber rules mean in the context of optimization approaches for mathematical models/what is specific to this setting?" in order to avoid redundancy.

- In its current state, the paper is not very clean. It needs some polishing to be really convincing: clarifying definitions, improving the text, etc. Please see below for more details.

General:

- It is not entirely clear to me whether the paper is about benchmarking for optimization methods or benchmarking for approaches involving an optimization step. These are two different issues.

- The distinction between pitfalls and guidelines (that allow avoiding pitfalls) is not very clear to me. The pitfalls (or at least some of them) could be re-formulated in terms of guidelines.

Specific:

- P1: The author presents real data analyses as a preferable alternative to simulations. But he does not say what are the advantages of simulations and the inconviences of real data analyses. In most real data analyses, the true values of the parameters are usually unknown, where as they are known for simulated data. Later in the manuscript, he says that this general rule does not hold in the investigated context. This discussion should appear earlier in the manuscript (the first time simulations are compared with real data analyses).

- P2: "The impact of co-factors can be limited by proper study designs." Firstly, I would like to know a bit more about these study designs. Secondly, I am not sure that the impact itself can be *minimized*. It can certainly be taken into account, reflected, etc, but minimized?

- P2: Cofactors are not properly defined. I realized this problem when, in P3, the datasets and the models are considered as cofactors. Please be more precise when defining such important concepts.

- G4: In a paper handling benchmarking for a specific type of methods, more details could be given here.

Typos:
- Please avoid referencing such as "as shown in [25]" or "[30] showed that".
- p2: non-linear on the model parameters -> non-linearly
- p2: "yield to" -> "yield" or "lead to"
- p3: "utilize restrict to": remove one of the two verbs.
- p6: "there is urgent need for benchmark problems": please reformulate (there is never "need for problem"...).

Please check the bibliography carefully. For example:
- All words included in journal names should begin with a capital letter.
- Ref. 5 is incomplete.
- Refs. 10 and 11 is incomplete.
- Provide links for Refs 23 and 24.
- etc etc etc. Please check!

**Reviewer 2**

**Is the topic of the article timely and of interest to a wide range of readers?**

Yes

**Is the article well written and presented in a logical way?**

Yes

**Do the authors cover the relevant literature in an accurate and balanced way?**

Yes

**Do the authors provide a useful synthesis of the topic?**

Yes

**Do the authors provide insightful discussion of the future directions for the field?**

Yes: The whole article is a discussion of the pitfalls and the future directions for application of optimization approaches for fitting mathematical models in systems biology.

**Do any tables, figures or boxes that are present enhance the manuscript?**

Yes

**Would the manuscript benefit from any additional tables, figures or boxes?**

No: I think that presented figures well demonstrate the points presented in the manuscript.

**Comments to author:**

The review article by Clemens Kreutz describes current challenges and offers guides for benchmarking of optimization approaches for fitting of mathematical models in systems biology. The subject of the article is interesting and the manuscript is well written. However, I find it a little bit too technical and therefore, in the present form, addressing a limited spectrum of readers. For example, some of the methods (e.g., multiple shooting) are referenced and discussed but not explained. It would help a non-specialist to have one-two sentence description of such terms. Therefore, I would suggest a more elaborate description of the 'Existing approaches and benchmark studies'. It is obvious that the Author is an expert in the field and a more biology-oriented reader could benefit a lot reading this part of the review.

Below, I am providing more detailed comments, but in general I would like to ask to make the manuscript language less technical. For example, I feel that the fragment "[...] a hybrid metaheuristic combining deterministic gradient-based optimization with a global scatter search metaheuristic" might be hard to digest by a biology-oriented reader.

Detailed comments:
1. In the third paragraph of Introduction the Author writes about absence of high-performing software implementations for parameter estimation. However, at the beginning of the third section ("Existing approaches...") the Author writes about "multitude of methods". Am I missing something or this is a contradictory statement?
2. In the second paragraph of the second section ("Why is reliable...") the Author states: 'the amount of available experimental data for parameter estimation is always limited'. I think that this sentence requires more elaborate explanation, especially in the context of the novel, currently available high-throughput technologies.
3. I do not understand first sentence from point (b) in the second section. The same concerns the point (d) from the same section. Please consider revising and / or elaborating.
4. In the fourth section ("Pitfals of benchmark...") the first sentence is not clear to me.
6. I do not agree with the points stated in "P1: Unrealistic setup". In many cases, because of lack of information, one has to use simulated data to precisely estimate the performance of the tested tools. Additionally, configuration parameters should be adjusted based on the performance of the method.
7. Concerning the cofactors ("P2: Ignoring cofactors"), I have again some double thoughts. In my opinion, many of the listed parameters should not be treated separately. Rather, they define gross characteristic of a tested tool — if a developer decides for a certain strategy (involving many of the listed factors), they can be assessed in a combined way.
8. The selection of case studies ("P3: Performing only case studies") was already mentioned in previous point (P2). This is the most obvious and common to any benchmarking study problem.
9. Finally, because of the recent publication on general guidelines for benchmark studies I would suggest to shorten the final chapter of the manuscript ("Guidelines for benchmark..."). In majority, this part of the text is coherent with the information provided by the Weber et al. paper.

**Authors Response**

**Point-by-point responses to the reviewers’ comments:**

Reviewer reports:

Reviewer #1: This paper handles an important topic and fits well into the current "benchmarking wave" in Genome Biology. I however have two major concerns:

- The proposed rules are, as admitted by the author himself, strongly related with the rules presented in the recent paper by Weber et al. Some repetition may be ok, but here I feel that this is too much. A possibility to avoid repetitions would be to generally formulate the current paper in the vein of "what do the Weber rules mean in the context of optimization approaches for mathematical models/what is specific to this setting?" in order to avoid redundancy.

*> Following the reviewer’s suggestion, the redundancy has been strongly reduced by rephrasing our guidelines. As suggested, we now stronger focus on interpretation of the Weber rules in the context of optimization. Moreover, in the new version of the review, this is explicitly emphasized in the following way:
>
> „Here, we concretize what those general suggestions mean for benchmarking of parameter optimization approaches and discuss their relevance, feasibility and differences in our context.“
>
> The previous guideline G9 (“future extensibility”) was removed, rephrased and included into guideline G8 (“publication and reproducibility”).
>
> Despite the concerns of the reviewer, I think that a small degree of coincidence is helpful in order to maintain a consistent picture covering both publications (especially since the Weber paper was also published in Genome Biology).
>
> For revision purpose, a file is attached where similar aspects (red color) as the Weber paper and optimization-specific issues (green color) are highlighted.
>*

- In its current state, the paper is not very clean. It needs some polishing to be really convincing: clarifying definitions, improving the text, etc. Please see below for more details.

*> The text has been strongly improved. The term cofactor has been entirely replaced by “covariate” including comprehensive definition. Moreover, several explanations of technical terms have been added.
>
> For revision purpose, a version of the manuscript has been submitted indicating the changes between the previous and the current version.
>*

General:

- It is not entirely clear to me whether the paper is about benchmarking for optimization methods or benchmarking for approaches involving an optimization step. These are two different issues.

*> We clarified this point by changing the text at several points, e.g. in the introduction, in section “Existing approaches…”, pitfall P2, and by rewriting the caption of Fig. 1. Moreover, the title has been changed slightly.
>*

- The distinction between pitfalls and guidelines (that allow avoiding pitfalls) is not very clear to me. The pitfalls (or at least some of them) could be re-formulated in terms of guidelines.

*> As argued by the reviewer, there is a relationship between pitfalls and guidelines. However, this is not necessarily one by one. Multiple guidelines might be required to prevent a pitfall. Conversely, a single guideline can help to prevent several pitfalls.
>
> In my manuscript, I termed serious problems and frequently made mistakes as pitfalls and a rather detailed discussion is provided. In contrast, guidelines are as precise and short as possible and formulated as rules. Both terms as well as their relationship are now clarified in the revised version.
>*

Specific:

- P1: The author presents real data analyses as a preferable alternative to simulations. But he does not say what are the advantages of simulations and the inconviences of real data analyses. In most real data analyses, the true values of the parameters are usually unknown, where as they are known for simulated data. Later in the manuscript, he says that this general rule does not hold in the investigated context. This discussion should appear earlier in the manuscript (the first time simulations are compared with real data analyses).

*> The discussion concerning simulated data has been moved to an earlier point in the manuscript (pitfall P1). Moreover, the discussion about advantages and disadvantages of simulations has been extended and improved (see pitfall P1 and guideline G3).
>*

- P2: "The impact of co-factors can be limited by proper study designs." Firstly, I would like to know a bit more about these study designs. Secondly, I am not sure that the impact itself can be *minimized*. It can certainly be taken into account, reflected, etc, but minimized?

*> Study designs are now described in more detail and an example is made.
> Moreover, the term “minimized” has been replaced by “reduced”.
>*

- P2: Cofactors are not properly defined. I realized this problem when, in P3, the datasets and the models are considered as cofactors. Please be more precise when defining such important concepts.

*> Thanks a lot for this hint. I decided to replace the term “cofactor” by “covariate” which is a much more common term. Moreover, “covariate” better covers continuous, i.e. non-factorial effects. In addition, a definition of the term “covariates” is now provided.
>*

- G4: In a paper handling benchmarking for a specific type of methods, more details could be given here.

*> In G4, the types of numerical tasks are now explicitly listed. Moreover, it is now referred to Tab. 1 in order to better illustrate configuration parameters. Moreover, an example for a performance loss is mentioned.
>*

Typos:
- Please avoid referencing such as "as shown in [25]" or "[30] showed that".
*> Done.*

- p2: non-linear on the model parameters -> non-linearly
*> Done.*

- p2: "yield to" -> "yield" or "lead to"
*> Done.*

- p3: "utilize restrict to": remove one of the two verbs.
*> Done.*

- p6: "there is urgent need for benchmark problems": please reformulate (there is never "need for problem"...).
*> Done*

Please check the bibliography carefully. For example:
- All words included in journal names should begin with a capital letter.
*> Done.*

Unfortunately, latexdiff seems to be not fully compatible with the latex style files for Genome Biology. There it was not possible to correctly highlight changes in the title, in section headings, as well as in the literature references.

- Ref. 5 is incomplete.
*> Done.*

- Refs. 10 and 11 is incomplete.
*> Done.*

- Provide links for Refs 23 and 24.
*> Done.*

- etc etc etc. Please check!
*> Done.*

Reviewer #2: The review article by Clemens Kreutz describes current challenges and offers guides for benchmarking of optimization approaches for fitting of mathematical models in systems biology. The subject of the article is interesting and the manuscript is well written. However, I find it a little bit too technical and therefore, in the present form, addressing a limited spectrum of readers. For example, some of the methods (e.g., multiple shooting) are referenced and discussed but not explained. It would help a non-specialist to have one-two sentence description of such terms. Therefore, I would suggest a more elaborate description of the 'Existing approaches and benchmark studies'. It is obvious that the Author is an expert in the field and a more biology-oriented reader could benefit a lot reading this part of the review.

The revised version of our manuscript contains more elaborate descriptions and explanations of technical terms and existing approaches. The following terms are now described: multiple shooting, trust region, hybrid methods, and finite differences, covariate).
Moreover, the mathematical term “manifold” has been replaced twice. The terms “cofactor” and “Hessian” have been replaced entirely.


Below, I am providing more detailed comments, but in general I would like to ask to make the manuscript language less technical. For example, I feel that the fragment "[...] a hybrid metaheuristic combining deterministic gradient-based optimization with a global scatter search metaheuristic" might be hard to digest by a biology-oriented reader.

*> The whole paragraph has been rephrased including this fragment. The reviewer’s suggestions below were considered.
>*

Detailed comments:
1. In the third paragraph of Introduction the Author writes about absence of high-performing software implementations for parameter estimation. However, at the beginning of the third section ("Existing approaches...") the Author writes about "multitude of methods". Am I missing something or this is a contradictory statement?

*> Fitting ODE models requires the combination of several generic tasks. For in these individual tasks, there are many methods. But it is difficult to combine these generic algorithms in a way that yield robust and high performing solutions for fitting of ODE models. These aspects are now explained in detail.*


2. In the second paragraph of the second section ("Why is reliable...") the Author states: 'the amount of available experimental data for parameter estimation is always limited'. I think that this sentence requires more elaborate explanation, especially in the context of the novel, currently available high-throughput technologies.

*> It is now explained that data is limited in terms of number of experimental conditions (i.e. time points, genetic perturbations, …).*

3. I do not understand first sentence from point (b) in the second section. The same concerns the point (d) from the same section. Please consider revising and / or elaborating.
*> Both points have been revised.*

4. In the fourth section ("Pitfals of benchmark...") the first sentence is not clear to me.
*> The respective sentence has been rephrased.*

6. I do not agree with the points stated in "P1: Unrealistic setup". In many cases, because of lack of information, one has to use simulated data to precisely estimate the performance of the tested tools.

*> The relevance of simulated data is very special for benchmark studies of optimization methods. The text in P1 and G3 has been strongly improved to properly explain these aspects and for emphasizing the special setting.
>*

Additionally, configuration parameters should be adjusted based on the performance of the method.

*> I’m not sure how exactly this is meant. Of course, configuration parameters have to be chosen properly and with the intention to improve the performance. However, this is hardly feasible for optimization-based fitting in real world settings and would require substantial computational efforts, i.e. something like a project-specific benchmark study for properly choosing configuration parameters.
>
>
> In a benchmark study that intends to derive generally valid conclusions, it has to be clearly stated, whether performance is assessed for default configurations, or after a clearly defined configuration procedure. In that case, however, the computational efforts of such a configuration procedure have to be counted as additional computational effort, e.g. in terms of runtime.
>
> The choice of configuration parameters is now discussed in more detail in the manuscript, e.g. in P1, P2. We now clearly emphasize that in
real applications, there is *only limited* information available for tuning of configuration parameters.
>*


7. Concerning the cofactors ("P2: Ignoring cofactors"), I have again some double thoughts. In my opinion, many of the listed parameters should not be treated separately. Rather, they define gross characteristic of a tested tool — if a developer decides for a certain strategy (involving many of the listed factors), they can be assessed in a combined way.

On the one hand, I agree. The listed parameters should not be treated separately and comprehensive strategies involving many listed parameters can be assessed.

*> On the other hand, investigating and understanding the general impact of covariates is also a major goal. Moreover, restricting to comprehensive strategies involving all decisions would limit the scope of a benchmark study to a special case and the impact of small deviations from the chosen strategy remains unclear. This demands for benchmarking of combinations which is infeasible.
>
> I think, this discussion corresponds to the classical variable selection issue in the case unknown order of the interactions. Evaluating the full search space is optimal but infeasible. Therefore, one has to investigate the interaction structure. Such a discussion, however, is not adequate for this review in Genome Biology.
>
> The whole paragraph has been rephrased in order to clarify this aspect.
>*

8. The selection of case studies ("P3: Performing only case studies") was already mentioned in previous point (P2). This is the most obvious and common to any benchmarking study problem.

*> Yes. P2 has been rephrased and repeating information has been removed.
>*

9. Finally, because of the recent publication on general guidelines for benchmark studies I would suggest to shorten the final chapter of the manuscript ("Guidelines for benchmark..."). In majority, this part of the text is coherent with the information provided by the Weber et al. paper.

*> The presentation of the guidelines has been partly rephrased and shortened. Redundancies with Weber et al. have been reduced up to a minimal level. For revision purpose, a file is attached where similar aspects (red color) as the Weber paper and optimization-specific issues (green color) are highlighted.
>*

**Second round of review**

**Reviewer 1**

The author nicely addressed most of my concerns. Here are some remaining issues.

- On P3: this paragraph mixes the choice of data examples and the choice of methods. These are two completely different aspects. Please clarify.
- The new terminology "covariate" is ok, but it seems that they are sometimes also denoted as "parameters", e.g., in G4. Is there a difference between these two terms? Please clarify.
- G7: "Benchmark studies should provide clear recommendation rules": I think this should be formulated more cautiously. In some cases, it is difficult to formulate clear recommendations because the results are not clear-cut. Formulating too definitive recommendations in such a case may be a mistake.
- The conclusion needs some polishing. While the author considerably improved the rest of the text, the conclusion still includes approximative/awkward statements.

- p2: "as model components Moreover". Dot is missing.
- p2: "all mathematical calculations requiring x(t) an explicit form": "in" is missing?
- p4: "the performance comparison of generic optimization approaches as core of the fitting process are usually of primary interest." are -> is.

**Reviewer 2**

The subject of the paper is of interest to the Systems Biology community and, in particular, the readers of Genome Biology. The paper is well written, striking a nice balance between the need to provide technical details and readability; I feel that the reader less interested in technical details may easily skim over the parts that are less accessible to get the gist of what is recommended. I have still noticed a few problems in the writing (mostly typos/grammar):
1.     LS and MLE; it might be of interest to note that these are linked.
2.     Phenomenological vs. mechanistic models; it might be of interest to note that many SB models need to balance the two approaches.
3.     “parameters and dynamic vs.”: I think something is missing in this sentence.
4.     problems  with singular/plural: “such a wrong models”, “assessment by distance…are”, “a set of benchmark problems has”, “general guidelines for benchmark…has”,
5.     unnecessary commas: “methods, how”, “stated, whether”, “both, convergence”
6.     “guidelines presented in the following”->”below”
7.     Awkward, rephrase: “Nevertheless, it should be aspired to include as many approaches as possible.” (In general, there is a lot of passive voice in this paper, but this sentence is particularly awkward).
8.     Rephrase: “the number of available benchmark problems is up to now strongly limited.”
9.     “simulated data only has”->”has only”?
10.     “only fits”->”only the fits”
11.     “We presented” (and in other places): there is only one author of this paper
12.     Figure 3 legend: “the same objective function”->”value of the objective function”?

**Authors Response**

**Point-by-point responses to the reviewers’ comments:**

Comment

*Response*

**Reviewer 1**

The author nicely addressed most of my concerns. Here are some remaining issues.

- On P3: this paragraph mixes the choice of data examples and the choice of methods. These are two completely different aspects. Please clarify.

*> According the the reviewer’s suggestion, the respective paragraph has been rephrased.*

- The new terminology "covariate" is ok, but it seems that they are sometimes also denoted as "parameters", e.g., in G4. Is there a difference between these two terms? Please clarify.

*> The term „configuration parameter“ has been replaced by „hyperparameter“. Moreover, the respective sentence has been rephrased to clarify that the term „covariates“ comprises hyperparameters and software versions.*

- G7: "Benchmark studies should provide clear recommendation rules": I think this should be formulated more cautiously. In some cases, it is difficult to formulate clear recommendations because the results are not clear-cut. Formulating too definitive recommendations in such a case may be a mistake.

*> Done.*

- The conclusion needs some polishing. While the author considerably improved the rest of the text, the conclusion still includes approximative/awkward statements.

*> According to the reviewer’s suggestion, the conclusion section has been polished.*

- p2: "as model components Moreover". Dot is missing.

*> Done.*

- p2: "all mathematical calculations requiring x(t) an explicit form": "in" is missing?

*> Done.*

- p4: "the performance comparison of generic optimization approaches as core of the fitting process are usually of primary interest." are -> is.

*> Done.*

**Reviewer 2**

The subject of the paper is of interest to the Systems Biology community and, in particular, the readers of Genome Biology. The paper is well written, striking a nice balance between the need to provide technical details and readability; I feel that the reader less interested in technical details may easily skim over the parts that are less accessible to get the gist of what is recommended. I have still noticed a few problems in the writing (mostly typos/grammar):
1.     LS and MLE; it might be of interest to note that these are linked.

*> Done.*

2.     Phenomenological vs. mechanistic models; it might be of interest to note that many SB models need to balance the two approaches.

*> Done.*

3.     “parameters and dynamic vs.”: I think something is missing in this sentence.

*> The reviewer is right. The mistake has been corrected.*

4.     problems  with singular/plural: “such a wrong models”, “assessment by distance…are”, “a set of benchmark problems has”, “general guidelines for benchmark…has”,

*> The respective sentences were checked and corrected.*

5.     unnecessary commas: “methods, how”, “stated, whether”, “both, convergence”

*> Done.*

6.     “guidelines presented in the following”->”below”

*> Done.*

7.     Awkward, rephrase: “Nevertheless, it should be aspired to include as many approaches as possible.” (In general, there is a lot of passive voice in this paper, but this sentence is particularly awkward).

*> The respective sentence has been rephrased.*

8.     Rephrase: “the number of available benchmark problems is up to now strongly limited.”

*> Done.*

9.     “simulated data only has”->”has only”?

*> Done.*

10.     “only fits”->”only the fits”

*> Done.*

11.     “We presented” (and in other places): there is only one author of this paper

*> „We“ has been replaced by „I“ throught the manuscript. Moreover, I replaced „our“ by „my“ where this seemed more adequate.*

12.     Figure 3 legend: “the same objective function”->”value of the objective function”?

*> Done.*
